# A Risk Stratification Model for Predicting Overall Survival and Surgical Benefit in Triple-Negative Breast Cancer Patients With *de novo* Distant Metastasis

**DOI:** 10.3389/fonc.2020.00014

**Published:** 2020-01-24

**Authors:** Zheng Wang, Hui Wang, Xi Sun, Yan Fang, Shuang-Shuang Lu, Shu-Ning Ding, Xiao-Song Chen, Kun-Wei Shen

**Affiliations:** Comprehensive Breast Health Center, Ruijin Hospital, Shanghai Jiao Tong University School of Medicine, Shanghai, China

**Keywords:** triple-negative breast cancer, metastasis, nomogram, overall survival, therapeutic decision

## Abstract

**Background and Aims:** This research aimed to construct a novel model for predicting overall survival (OS) and surgical benefit in triple-negative breast cancer (TNBC) patients with *de novo* distant metastasis.

**Methods:** We collected data from the Surveillance, Epidemiology, and End Results (SEER) database for TNBC patients with distant metastasis between 2010 and 2016. Patients were excluded if the data regarding metastatic status, follow-up time, or clinicopathological information were incomplete. Univariate and multivariate analyses were applied to identify significant prognostic parameters. By integrating these variables, a predictive nomogram and risk stratification model were constructed and assessed with C-indexes and calibration curves.

**Results:** A total of 1,737 patients were finally identified. Patients enrolled from 2010 to 2014 were randomly assigned to two cohorts, 918 patients in the training cohort and 306 patients in the validation cohort I, and 513 patients enrolled from 2015 to 2016 were assigned to validation cohort II. Seven clinicopathological factors were included as prognostic variables in the nomogram: age, marital status, T stage, bone metastasis, brain metastasis, liver metastasis, and lung metastasis. The C-indexes were 0.72 [95% confidence interval [CI] 0.68–0.76] in the training cohort, 0.71 (95% CI 0.68–0.74) in validation cohort I and 0.71 (95% CI 0.67–0.75) in validation cohort II. Calibration plots indicated that the nomogram-based predictive outcome had good consistency with the recoded prognosis. A risk stratification model was further generated to accurately differentiate patients into three prognostic groups. In all cohorts, the median overall survival time in the low-, intermediate- and high-risk groups was 17.0 months (95% CI 15.6–18.4), 11.0 months (95% CI 10.0–12.0), and 6.0 months (95% CI 4.7–7.3), respectively. Locoregional surgery improved prognosis in both the low-risk [hazard ratio [HR] 0.49, 95% CI 0.41–0.60, *P* < 0.0001] and intermediate-risk groups (HR 0.55, 95% CI 0.46–0.67, *P* < 0.0001), but not in high-risk group (HR 0.73, 95% CI 0.52–1.03, *P* = 0.068). All stratified groups could prognostically benefit from chemotherapy (low-risk group: HR 0.50, 95% CI 0.35–0.69, *P* < 0.0001; intermediate-risk group: HR 0.34, 95% CI 0.26–0.44, *P* < 0.0001; and high-risk group: HR 0.16, 95% CI 0.10–0.25, *P* < 0.0001).

**Conclusion:** A predictive nomogram and risk stratification model were constructed to assess prognosis in TNBC patients with *de novo* distant metastasis; these methods may provide additional introspection, integration and improvement for therapeutic decisions and further studies.

## Introduction

Triple-negative breast cancer (TNBC) is a biologically invasive disease that accounts for ~15% of breast malignancies (1). Despite the rapid development of treatment methods such as surgery, chemotherapy and immunotherapy, TNBC is still the common cause for cancer-related deaths, mainly due to distant metastasis (2).

Cancer metastasis is a complicated process, involving several stages such as invasion of the extracellular matrix, epithelial-mesenchymal transition, angiogenesis, immune invasion, and distal colonization (3). Usually during the process of distant metastasis, cancer cells (seed) escape from the primary site and adapt to the distant microenvironment (soil), which can be mediated by the “seed and soil” interaction (4). Furthermore, distant target organs can be changed and prepared for the arrest and colonization of circulating cancer cells (5, 6). In terms of triple-negative breast cancer, several studies have indicated that different genes mediate tumor cell metastasis to either bone, lung, brain or liver tissues, resulting in organ-specific metastatic heterogeneity (7–10).

In the real world, metastatic TNBC is a heterogeneous neoplasm with diverse prognostic endings and can be influenced by demographic features, including age, race and marital status, as well as clinicopathological parameters (for example, tumor size, grade, and clinical treatment) (11–14). Different metastatic sites can also influence the survival outcomes of TNBC. For instance, visceral metastasis results in a poorer prognosis than bone metastasis (15). Thus, in consideration of these clinicopathological factors that may influence patient survival, it is vital to construct a comprehensive analytic model to accurately estimate the prognostic outcome of every patient. This predictive model can help physicians make therapeutic decisions and perform clinical trials.

In recent years, the nomogram has been considered a commonly viable predictive model for assessing prognostic outcome, especially in cancer patients (16–20). Several nomograms have been established for predicting the risk of recurrence, the benefit of radiation or the response to neoadjuvant chemotherapy in breast cancer (21–23). However, no nomogram has been developed for predicting the survival outcomes of TNBC patients diagnosed with *de novo* distant metastasis. Thus, in the present research, we intended to establish and validate a nomogram for the general distantly metastatic TNBC set.

## Materials and Methods

### Cohort Population and Data Processing

This was a retrospective study based on data from the Surveillance, Epidemiology, and End Results (SEER) database. In this study, case selection was conducted on the basis of the following inclusion and exclusion criteria.

Inclusion criteria: (1) pathological diagnosis was made between 2010 and 2016; (2) molecular subtype of triple-negative breast cancer; and (3) at least one distant site of *de novo* metastasis.

Exclusion criteria: (1) male breast cancer; (2) unknown metastatic status; (3) missing follow-up data; (4) incomplete clinicopathological information including race, marital status, grade, T/N stage and therapy.

### Statistical Analysis

We randomly assigned the patients enrolled from 2010 to 2014 into two cohorts, the training cohort and the validation cohort I, at a ratio of three to one, and we assigned the patients enrolled from 2015 to 2016 into the validation cohort II. Descriptive statistics were applied to summarize the clinicopathological features of the three cohorts. Overall survival (OS) was compared among different subgroups with Kaplan-Meier methods and log-rank tests. Further multivariate modeling was conducted to assess the independent predictive variables for survival. In consideration of potential competitive risk factors, breast cancer-specific survival (BCSS) was further analyzed with univariate and multivariate models. Cumulative mortality curves were generated to assess the impact of competitive mortality. Statistical significance was determined with a two-sided *P* < 0.05. We executed statistical analyses with SPSS 22.0.

Based on the data of the multivariate model, a nomogram was constructed with RMS and the SURVIVAL package in R software. We used 2-, 3-, and 5-years OS for analysis in the nomogram. One thousand bootstrap resamples were used to calculate C-indexes and generate calibration plots, which assessed the predictive accuracy of the nomogram. Furthermore, a risk stratification model was developed on the basis of each patient's total scores in the nomogram to divide all cases into three prognostic groups.

## Results

### Patient Characteristics

**Figure 1:**
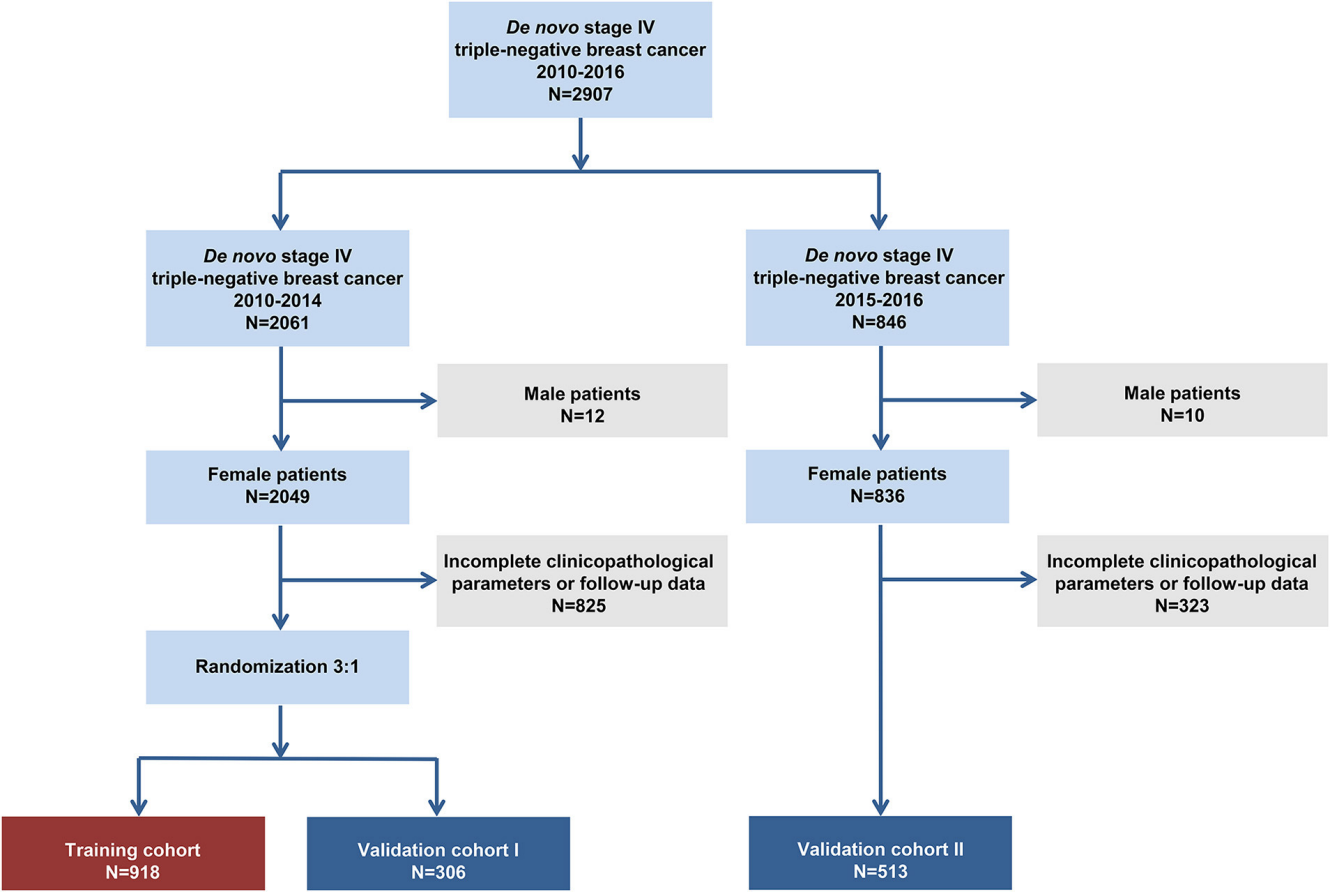
Patient selection flowchart.

The flowchart of the patient selection process is shown in Figure 1. In total, we included 1,737 patients based on the following criteria: 918 patients in the training set, 306 patients in validation set I and 513 patients in validation set II. The patients' baseline clinicopathological features and OS data within each subgroup are shown in Table 1. In the training set, 24.5% (225/918), 52.8% (485/918), and 22.7% (208/918) of the patients aged <50, 50–69, and ≥70, respectively. In addition, 9.9% (91/918), 29.5% (271/918), 19.6% (180/918), and 41.0% (376/918) of the patients had stage T1, T2 T3, and T4 tumors, respectively. Furthermore, 22.0% (202/918) of the patients had negative N stage and 78.0% (716/918) had positive N stage.

**Table 1:** Baseline clinicopathological characteristics of the included patients with initially diagnosed metastatic triple-negative breast cancer.

**Clinicopathological characteristics**	**Training set (*****N*** **=** **918)**		**Validation set I (*****N*** **=** **306)**		**Validation set II (*****N*** **=** **513)**	
	**No. of patients (%)**	**Median OS (95% CI)**	**No. of patients (%)**	**Median OS (95% CI)**	**No. of patients (%)**	**Median OS (95% CI)**
**Race**						
White	630 (68.6)	13.0 (11.9–14.1)	194 (63.4)	12.0 (10.0–14.0)	343 (66.9)	14.0 (12.4–15.6)
Black	236 (25.7)	12.0 (10.5–13.5)	88 (28.8)	12.0 (9.7–14.3)	125 (24.4)	11.0 (10.0–12.0)
OthersΔ	52 (5.7)	13.0 (8.0–18.0)	24 (7.8)	14.0 (11.1–16.9)	45 (8.8)	14.0 (11.3–16.7)
**Age**						
<50	225 (24.5)	15.0 (13.2–16.8)	74 (24.2)	14.0 (12.3–15.7)	120 (23.4)	15.0 (12.7–17.3)
50–69	485 (52.8)	13.0 (11.4–14.6)	164 (53.6)	13.0 (11.3–1.47)	257 (50.1)	14.0 (10.8–17.2)
≥70	208 (22.7)	8.0 (5.6–10.4)	68 (22.2)	9.0 (5.0–13.0)	136 (26.5)	10.0 (7.8–12.2)
**Marriage**						
Married	383 (41.7)	15.0 (13.4–16.6)	126 (41.2)	14.0 (12.1–15.9)	241 (47.0)	16.0 (12.9–19.1)
Unmarried	535 (58.3)	11.0 (9.8–12.2)	180 (58.8)	12.0 (10.3–13.7)	272 (53.0)	11.0 (9.9–12.1)
**Grade**						
I	12 (1.3)	13.0 (7.9–18.1)	1 (0.3)	/	7 (1.4)	/
II	155 (16.9)	13.0 (12.0–14.0)	50 (16.3)	13.0 (11.4–14.6)	96 (18.7)	13.0 (11.5–14.5)
III	751 (81.8)	11.0 (7.8–14.2)	255 (83.3)	12.0 (9.4–14.6)	410 (79.9)	13.0 (11.2–14.8)
**T stage**						
T1	91 (9.9)	16.0 (10.3–21.7)	30 (9.8)	14.0 (10.0–18.0)	52 (10.1)	17.0 (10.8–23.2)
T2	271 (29.5)	15.0 (13.0–17.0)	94 (30.7)	14.0 (12.0–16.0)	149 (29.0)	14.0 (10.3–17.7)
T3	180 (19.6)	14.0 (11.8–16.2)	57 (18.6)	13.0 (10.3–15.7)	104 (20.3)	13.0 (10.2–15.8)
T4	376 (41.0)	9.0 (7.6–10.4)	125 (40.8)	11.0 (9.1–12.9)	208 (40.5)	12.0 (9.8–14.2)
**N stage**						
Negative	202 (22.0)	13.0 (12.0–14.0)	47 (15.4)	13.0 (11.6–14.4)	105 (20.5)	14.0 (11.1–16.9)
Positive	716 (78.0)	11.0 (8.7–13.3)	259 (84.6)	11.0 (7.0–15.0)	408 (79.5)	13.0 (11.1–14.9)
**Bone metastasis**						
No	538 (58.6)	15.0 (13.4–16.6)	173 (56.5)	14.0 (12.5–15.5)	284 (55.4)	15.0 (13.4–16.6)
Yes	380 (41.4)	11.0 (9.6–12.4)	133 (43.5)	10.0 (7.7–12.3)	229 (44.6)	11.0 (8.8–13.2)
**Brain metastasis**						
No	835 (91.0)	13.0 (12.1–13.9)	271 (88.6)	13.0 (11.5–14.5)	460 (89.7)	14.0 (12.3–15.7)
Yes	83 (9.0)	6.0 (3.5–8.5)	35 (11.4)	7.0 (2.4–11.6)	53 (10.3)	6.0 (4.0–8.0)
**Liver metastasis**						
No	654 (71.2)	15.0 (13.6–16.4)	220 (71.9)	13.0 (11.6–14.4)	375 (73.1)	14.0 (12.1–15.9)
Yes	264 (28.8)	9.0 (7.3–10.7)	86 (28.1)	8.0 (4.2–11.8)	138 (26.9)	11.0 (6.5–15.5)
**Lung metastasis**						
No	533 (58.1)	13.0 (11.7–14.3)	199 (65.0)	14.0 (12.8–15.2)	306 (59.6)	14.0 (11.8–16.2)
Yes	385 (41.9)	12.0 (10.6–13.4)	107 (35.0)	10.0 (7.1–12.9)	207 (40.4)	12.0 (9.4–14.6)
**Chemotherapy**						
No	203 (22.1)	3.0 (2.2–3.8)	65 (21.2)	2.0 (1.4–2.6)	105 (20.5)	3.0 (1.7–4.3)
Yes	715 (77.9)	15.0 (13.8–16.2)	241 (78.8)	15.0 (13.8–16.2)	408 (79.5)	15.0 (13.4–16.6)
**Surgery**						
No	465 (50.7)	8.0 (6.9–9.1)	160 (52.3)	10.0 (7.8–12.2)	333 (64.9)	10.0 (8.5–11.5)
Yes	453 (49.3)	18.0 (16.5–19.5)	146 (47.7)	16.0 (12.7–19.3)	180 (35.1)	18.0 (14.3–21.7)

*ΔOthers include American Indian, AK Native, Asian, and Pacific Islander*.

*OS, Overall Survival; CI, Confidence Interval*.

In terms of the different metastatic sites, 41.4% (380/918), 9.0% (83/918), 28.8% (264/918), and 41.9% (385/918) of the patients had metastasis to the bone, brain, liver and lung, respectively, in the training set. The median overall survival time was 11.0 (95% CI 9.6–12.4), 6.0 (95% CI 3.5–8.5), 9.0 (95% CI 7.3–10.7), and 12.0 (95% CI 10.6–13.4) months for patients with bone, brain, liver and lung metastasis, respectively.

### Univariate and Multivariate Analyses for Prognosis

**Figure 2:**
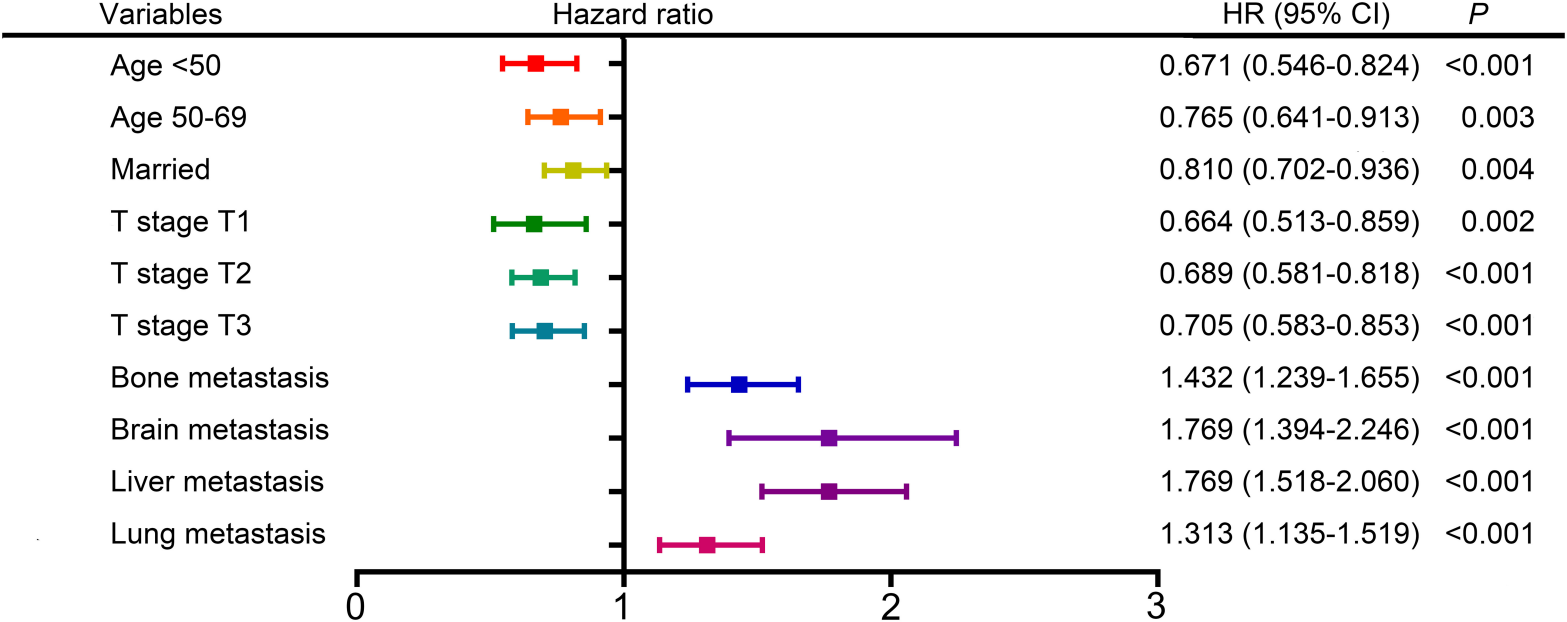
Forest plot showing the results of multivariate analysis for overall survival.

The following clinicopathological variables were found to be statistically significant factors for overall survival: age (<50: HR 0.671, 95% CI 0.546–0.824; 50–69: HR 0.765, 95% CI 0.641–0.913; ≥70 as a reference), marital status (married: HR 0.810, 95% CI 0.702–0.936; unmarried as a reference), T stage (T1: HR 0.664, 95% 0.513–0.859; T2: HR 0.689, 95% CI 0.581–0.818; T3: HR 0.705, 95% CI 0.583–0.853; T4 as a reference), bone metastasis (metastasis: HR 1.432, 95% CI 1.239–1.655; no metastasis as a reference), brain metastasis (metastasis: HR 1.769, 95% CI 1.394–2.246; no metastasis as a reference), liver metastasis (metastasis: HR 1.769, 95% CI 1.518–2.060; no metastasis as a reference), lung metastasis (metastasis: HR 1.313, 95% CI 1.135–1.519; no metastasis as a reference) (Table 2, Figure 2). Furthermore, univariate and multivariate analyses identified the same prognostic factors for breast cancer-specific survival (Supplementary Table 1, Supplementary Figure 1). Thus, we included all these prognostic factors for nomogram construction.

**Table 2:** Univariate and multivariate analyses for overall survival.

**Clinicopathological characteristics**	**Univariable analysis *P***	**Multivariable analysis**	
		**Hazard ratio (95% CI)**	***P***
Race	0.810		
White			
Black			
Others			
Age	0.001		0.001
<50		0.671 (0.546–0.824)	<0.001
50–69		0.765 (0.641–0.913)	0.003
≥70		Reference	
Marriage	<0.001		0.004
Married		0.810 (0.702–0.936)	0.004
Unmarried		Reference	
Grade	0.441		
I			
II			
III			
T stage	<0.001		<0.001
T1		0.664 (0.513–0.859)	0.002
T2		0.689 (0.581–0.818)	<0.001
T3		0.705 (0.583–0.853)	<0.001
T4		Reference	
N stage	0.249		
Negative			
Positive			
Bone metastasis	<0.001		<0.001
Yes		1.432 (1.239–1.655)	<0.001
No		Reference	
Brain metastasis	<0.001		<0.001
Yes		1.769 (1.394–2.246)	<0.001
No		Reference	
Liver metastasis	<0.001		<0.001
Yes		1.769 (1.518–2.060)	<0.001
No		Reference	
Lung metastasis	<0.001		<0.001
Yes		1.313 (1.135–1.519)	<0.001
No		Reference	

### Nomogram Construction and Validation

**Figure 3:**
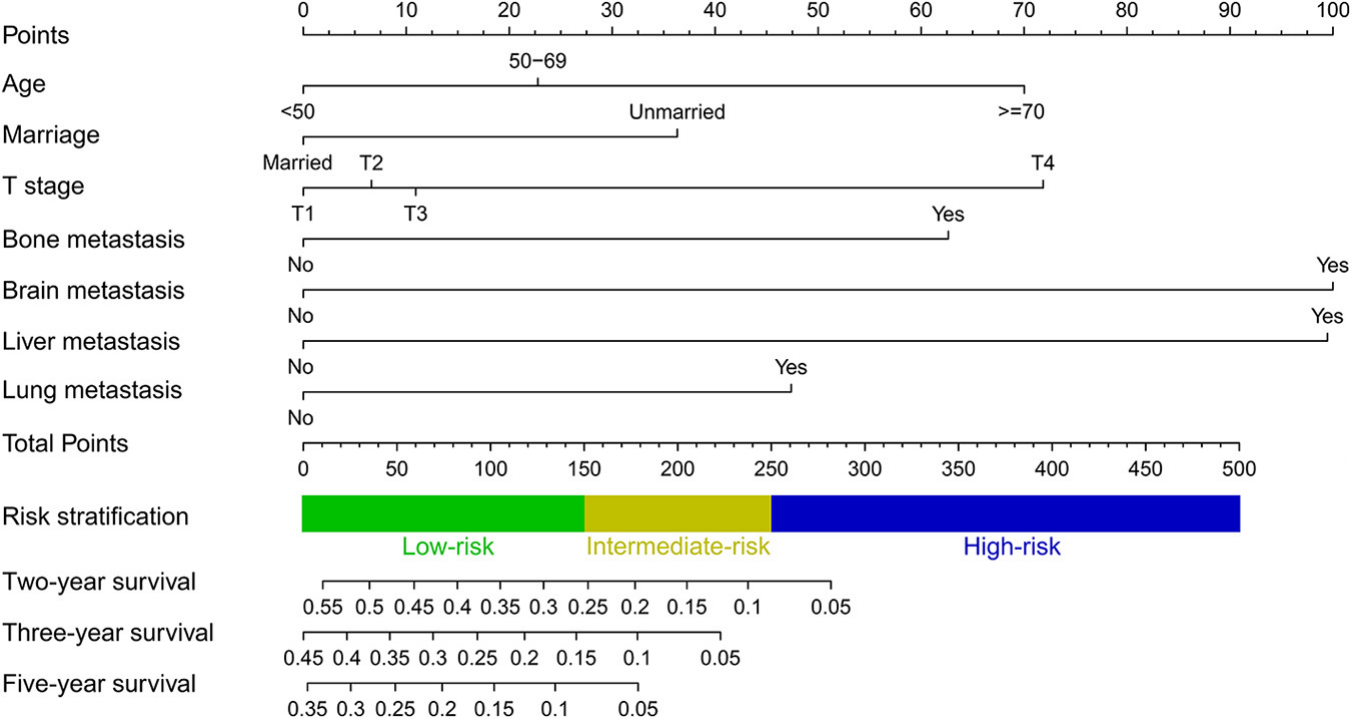
Nomogram for predicting 2-, 3- and 5-years overall survival in TNBC patients with *de novo* distant metastasis.

A predictive nomogram integrating seven independent risk factors for prognosis was constructed (Figure 3) and scores were assigned for the clinical variables in each subgroup (Table 3). Among all included variables, brain metastasis had a score of 100, followed by liver metastasis (score 99), T stage (T4: score 72; T3: score 11; T2: score 7), age (≥70: score 70; 50–69: score 23), bone metastasis (score 63), lung metastasis (score 47), and marital status (unmarried: score 36). The total score of an individual patient was obtained by adding all scores based on the patient's clinical variables. The likelihood of 2-, 3-, and 5-years OS could be obtained by drawing a straight line on the “total points” axis (Figure 3).

**Table 3:** Scores of clinical variables in each subgroup.

**Variables**	**Points**	**Variables**	**Points**
Age		Bone metastasis	
<50	0	No	0
50–69	23	Yes	63
≥70	70	Lung metastasis	
Marriage		No	0
Married	0	Yes	47
Unmarried	36	Liver metastasis	
T stage		No	0
T1	0	Yes	99
T2	7	Brain metastasis	
T3	11	No	0
T4	72	Yes	100

**Figure 4:**
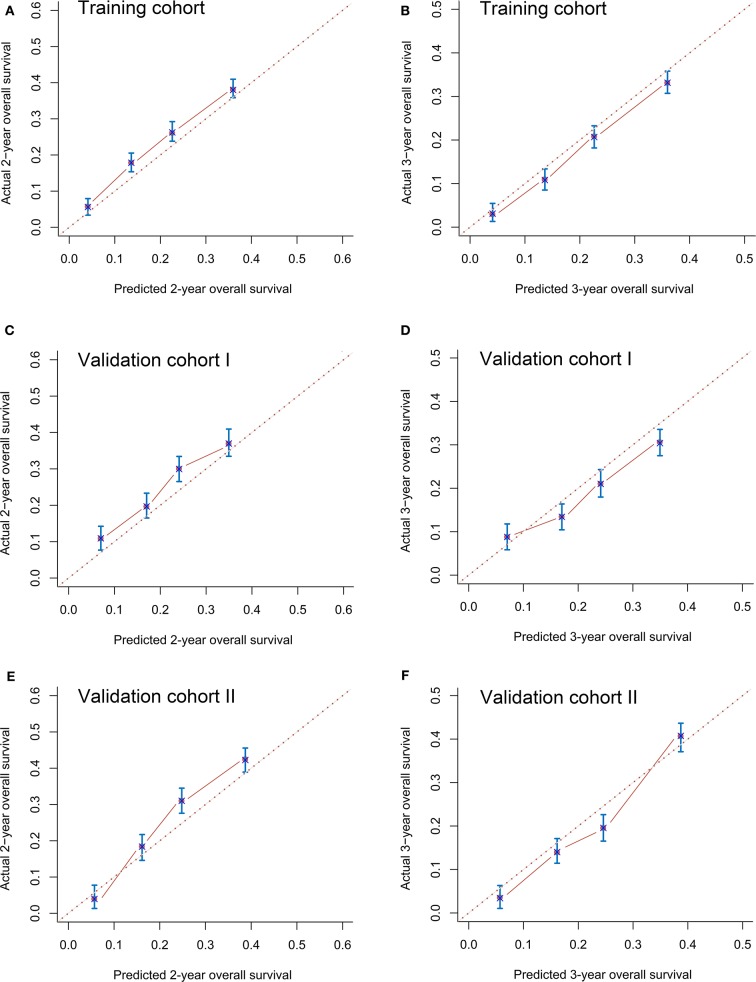
Calibration curves for predicting 2-years **(A)** and 3-years **(B)** overall survival in the training cohort, 2-years **(C)** and 3-years **(D)** overall survival in validation cohort I and 2-years **(E)** and 3-years **(F)** overall survival in validation cohort II.

The C-indexes in the training (0.72, 95% CI 0.68–0.76), validation I (0.71, 95% CI 0.68–0.74), and validation II (0.71, 95% CI 0.67–0.75) cohorts suggested acceptable predictive accuracy of the model. The calibration plots in the training set suggested that the predictive outcome had good agreement with the recorded survival results (Figures 4A,B). The calibration curves in validation sets I and II also showed that the nomogram-based predictive outcome had good consistency with the recoded prognosis results (Figures 4C–F).

### Risk Stratification Model

**Figure 5:**
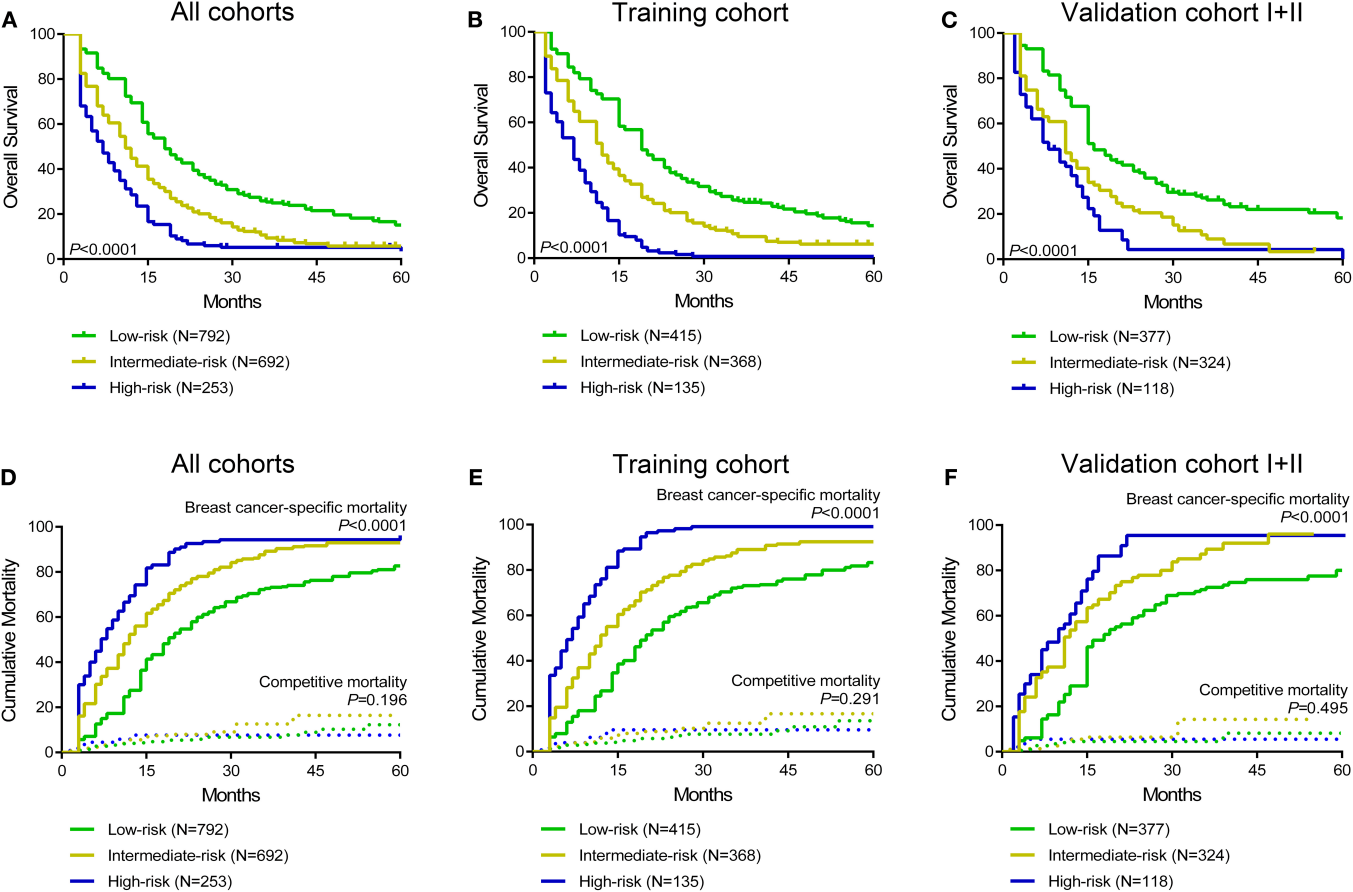
Kaplan curves of low-, intermediate- and high-risk groups in all cohorts **(A)**, the training cohort **(B)**, and validation cohort I+II **(C)**. Cumulative breast cancer-specific and competitive mortality curves stratified by risk groups in all cohorts **(D)**, the training cohort **(E)**, and validation cohort I+II **(F)**.

Moreover, a risk stratification model was generated on the basis of each patient's total scores from the nomogram to divide all patients into three prognostic groups. According to the risk stratification model, all the patients were stratified into three groups: low-risk group (792/1,737, 45.6%; total score <150), intermediate-risk group (692/1,737, 39.8%; total score 150–249), and high-risk group (253/1,737, 14.6%; total score ≥ 250) (Figure 3). In all cohorts, the median overall survival time in the low-, intermediate- and high-risk groups was 17.0 months (95% CI 15.6–18.4), 11.0 months (95% CI 10.0–12.0), and 6.0 months (95% CI 4.7–7.3), respectively. The Kaplan-Meier methods indicated that the risk stratification model could accurately differentiate survival in the three prognostic groups (Figures 5A–C). Cumulative mortality curves were generated to assess the impact of competitive events. There was no significant difference with regard to competitive mortality in all cohorts (*P* > 0.05) (Figures 5D–F), indicating that the primary outcome in this research was not affected by the potential competitive risk factors.

### Survival Benefit of Surgery and Systemic Therapy in Stratified Risk Groups

**Figure 6:**
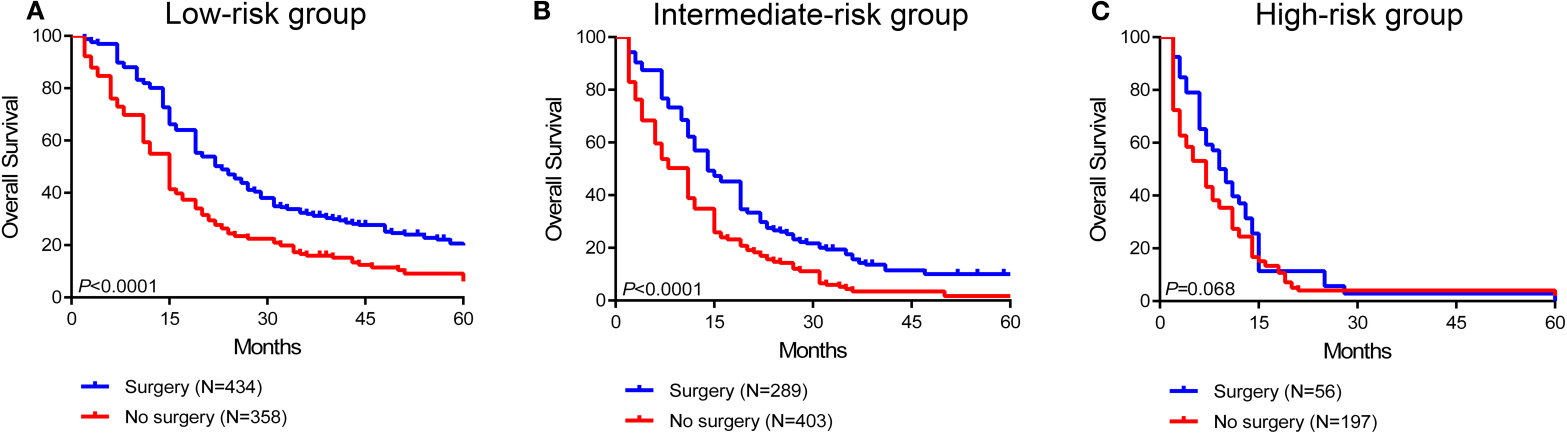
Survival benefit of surgery in the low-risk **(A)**, intermediate-risk **(B)**, and high-risk **(C)** groups.

**Figure 7:**
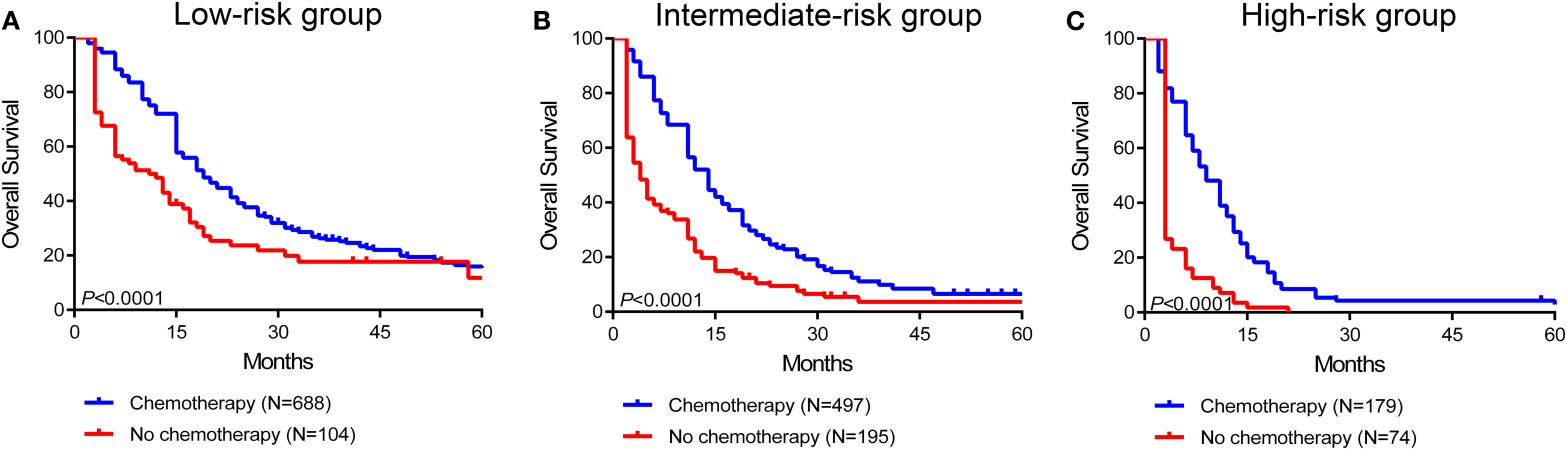
Survival benefit of chemotherapy in the low-risk **(A)**, intermediate-risk **(B)**, and high-risk **(C)** groups.

To further assess the survival benefit of surgery, Kaplan-Meier curves were generated in the stratified risk groups. The results showed that surgery could prolong overall survival in both the low- and intermediate-risk groups (low-risk group: HR 0.49, 95% CI 0.41–0.60, *P* < 0.0001; intermediate-risk group: HR 0.55, 95% CI 0.46–0.67, *P* < 0.0001) (Figures 6A,B). However, surgery did not significantly improve prognosis in the high-risk group (HR 0.73, 95% CI 0.52–1.03, *P* = 0.068) (Figure 6C). In terms of systemic therapy, all stratified groups could prognostically benefit from chemotherapy (low-risk group: HR 0.50, 95% CI 0.35–0.69, *P* < 0.0001; intermediate-risk group: HR 0.34, 95% CI 0.26–0.44, *P* < 0.0001; high-risk group: HR 0.16, 95% CI 0.10–0.25, *P* < 0.0001) (Figures 7A–C).

## Discussion

In the present study, a nomogram was conducted and validated for predicting survival outcomes in distantly metastatic TNBC patients. We finally included 1,737 patients and identified seven demographic and clinicopathological features as prognostic factors including age, marital status, T stage, and bone/brain/liver/lung metastasis. Further C-index assessment and calibration curves suggested that the nomogram had optimal predictive accuracy. Moreover, a risk stratification model was generated on the basis of each patient's total scores from the nomogram and the survival benefits of therapeutic choices were analyzed in the classified risk groups.

To the best of knowledge, this is the first large-cohort, comprehensive retrospective study that has developed a predictive nomogram for the prognosis of TNBC patients with distant organ metastasis. Our prognostic model can be feasibly applied in clinical practice to predict the survival probability of each individual patient, and remind doctors of the expected benefits of different treatments. Furthermore, the newly established risk stratification system recognizes high-risk patients who need additional adjuvant therapies. Follow-up period can be narrowed for timely adjustment of treatment protocols in the high-risk subgroups. In the meantime, these high-risk patients can also be encouraged to take part in ongoing clinical trials for novel drugs. Moreover, this predictive tool is useful for the guidance of controlling confounding bias in research design, especially in those regarding overall survival as primary endpoints. In brief, we believe that patients enrolled for nomogram construction represent the majority of metastatic TNBC patients, which guarantees the translational value of this predictive model in real situations.

In our findings, demographic features (age and marital status) and clinicopathological variables (T stage and bone/brain/liver/lung metastasis) were independent prognostic factors, results that were consistent with previous publications (11, 24, 25). Among all these distal metastatic sites, brain metastasis was the key factor with the poorest prognosis, followed by liver, lung and bone metastasis. A previous large-cohort study considered breast cancer patients as a whole population and showed a similar trend in terms of the influence of different distant metastatic sites on patient survival (13).

The standard treatment for TNBC patients with *de novo* distant metastasis usually consists of palliative systemic therapies such as chemotherapy. The survival benefit of locoregional resection remains controversial. A multicenter, phase III, randomized, controlled trial MF07-01 indicated that locoregional treatment could improve 5-years survival in *de novo* stage IV breast cancer patients (26). A recently published multicentric retrospective study in France indicated that locoregional treatment improved overall survival in breast cancer patients with synchronous metastasis, especially in patients with the molecular subtype of HR-positive/HER2-negative and HER2-positive (27). Another retrospective study in Chinese patients showed that surgical removal of the primary tumor could improve the prognosis of patients with bone metastasis alone (28). Importantly, surgery can offer solid pathological evidence for molecular classification, can alleviate clinical symptoms and can reduce tumor burden. However, not all patients can obtain a survival benefit from locoregional therapy. The ABCSG-28 trial did not indicate a survival benefit for locoregional surgery in *de novo* metastatic breast cancer (29). Another open-labeled randomized controlled trial in India also identified that breast operations could not prolong survival in patients with primary metastasis (30). Thus, personal demographic and clinicopathological parameters need to be considered carefully to make a therapeutic decision for each patient. It is vital to construct a risk stratification model integrating all these parameters to precisely identify those patients who can prognostically benefit from locoregional resection. Notably, in our established model, surgery could only improve the survival outcome in low- and intermediate-risk groups, but not in high-risk groups, which provided more accurate information for therapeutic decisions.

To our knowledge, this research is among the innovative studies that have conducted a predictive nomogram for general metastatic TNBC patients. However, there may be several limitations in the present research. The first may be the retrospective nature of SEER-based research. Second, information about some potential prognostic parameters, such as the Eastern Cooperative Oncology Group (ECOG) performance status score, the detailed chemotherapy protocol and the multigene signature assessment, were not provided in the database (31–33). In addition, the database only included information on *de novo* distant metastasis. Some patients may have developed metachronous metastasis during follow-up which is unknown from the database. Last, only the patients diagnosed from 2010 to 2016 were ultimately enrolled for analysis, since distant metastatic locations and molecular classification were recorded from 2010 in the SEER database. Additionally, the majority of enrolled patients were Caucasian and black, so the nomogram needs to be validated in external cohorts, especially in Asian patients. Thus, we suggest further prospective studies be performed and that more prognostic variables be considered to improve our predictive model.

In summary, a novel predictive nomogram and risk stratification model were conducted for predicting individual survival in TNBC patients with *de novo* distant metastasis. This prognostic model may help clinical physicians make better decisions and may help in the design of future prospective studies.

## Data Availability Statement

The datasets analyzed for this study can be found in the SEER database (https://seer.cancer.gov/).

## Ethics Statement

This research was based on the publicly available data from the SEER database and the data-use agreement was assigned. Patients' informed consent was not required because no direct interaction with patients was performed and no personal identification was applied in this study. In addition, this research was conducted in compliance with the Declaration of Helsinki.

## Author Contributions

ZW, HW, and X-SC designed this study. ZW, XS, and YF performed the search and collected data. S-SL and S-ND rechecked data. ZW and XS performed analysis and wrote the manuscript. X-SC and K-WS helped to revise the manuscript. All authors approved the final version of the manuscript.

### Conflict of Interest

The authors declare that the research was conducted in the absence of any commercial or financial relationships that could be construed as a potential conflict of interest.

## Supplementary Material

Supplementary Figure 1 Forest plot showing the results of multivariate analysis for breast cancer-specific survival.

Supplementary Table 1 Univariate and multivariate analyses for breast cancer-specific survival.

## Abbreviations

TNBC: Triple-Negative Breast Cancer
OS: Overall Survival
BCSS: Breast Cancer-Specific Survival
HR: Hazard Ratio
CI: Confidence Interval
SEER: Surveillance, Epidemiology, and End Results
ECOG: Eastern Cooperative Oncology Group.

## References

[1] Dent R Trudeau M Pritchard KI Hanna WM Kahn HK Sawka CA. Triple-negative breast cancer: clinical features and patterns of recurrence. Clin Cancer Res. (2007) 13:4429–34. 10.1158/1078-0432.CCR-06-3045 17671126

[2] Harbeck N Gnant M. Breast cancer. Lancet. (2017) 389:1134–50. 10.1016/S0140-6736(16)31891-8 27865536

[3] Fidler IJ. The pathogenesis of cancer metastasis: the ‘seed and soil' hypothesis revisited. Nat Rev Cancer. (2003) 3:453–8. 10.1038/nrc1098 12778135

[4] de Groot AE Roy S Brown JS Pienta KJ Amend SR. Revisiting seed and soil: examining the primary tumor and cancer cell foraging in metastasis. Mol Cancer Res. (2017) 15:361–70. 10.1158/1541-7786.MCR-16-0436 28209759 PMC5380470

[5] Chambers AF Groom AC MacDonald IC. Dissemination and growth of cancer cells in metastatic sites. Nat Rev Cancer. (2002) 2:563–72. 10.1038/nrc865 12154349

[6] Gupta GP Massague J. Cancer metastasis: building a framework. Cell. (2006) 127:679–95. 10.1016/j.cell.2006.11.001 17110329

[7] Kang Y Siegel PM Shu W Drobnjak M Kakonen SM Cordón-Cardo C. A multigenic program mediating breast cancer metastasis to bone. Cancer Cell. (2003) 3:537–49. 10.1016/S1535-6108(03)00132-6 12842083

[8] Minn AJ Gupta GP Siegel PM Bos PD Shu W Giri DD. Genes that mediate breast cancer metastasis to lung. Nature. (2005) 436:518–24. 10.1038/nature03799 16049480 PMC1283098

[9] Bos PD Zhang XH Nadal C Shu W Gomis RR Nguyen DX. Genes that mediate breast cancer metastasis to the brain. Nature. (2009) 459:1005–9. 10.1038/nature08021 19421193 PMC2698953

[10] Tabariès S Dong Z Annis MG Omeroglu A Pepin F Ouellet V. Claudin-2 is selectively enriched in and promotes the formation of breast cancer liver metastases through engagement of integrin complexes. Oncogene. (2011) 30:1318–28. 10.1038/onc.2010.518 21076473

[11] Chen MT Sun HF Zhao Y Fu WY Yang LP Gao SP. Comparison of patterns and prognosis among distant metastatic breast cancer patients by age groups: a SEER population-based analysis. Sci Rep. (2017) 7:9254. 10.1038/s41598-017-10166-8 28835702 PMC5569011

[12] Wang K Shi Y Li ZY Xiao YL Li J Zhang X. Metastatic pattern discriminates survival benefit of primary surgery for *de novo* stage IV breast cancer: a real-world observational study. Eur J Surg Oncol. (2019) 45:1364–72. 10.1016/j.ejso.2019.02.013 30837102

[13] Xiao W Zheng S Yang A Zhang X Zou Y Tang H. Breast cancer subtypes and the risk of distant metastasis at initial diagnosis: a population-based study. Cancer Manage Res. (2018) 10:5329–38. 10.2147/CMAR.S176763 30464629 PMC6225920

[14] Mandó P Rizzo M de la Puente CP Maino M Ponce C Pombo MT. High histologic grade and high Ki-67 expression predict phenotypic alterations in node metastasis in primary breast cancers. J Breast Cancer. (2017) 20:170–5. 10.4048/jbc.2017.20.2.170 28690653 PMC5500400

[15] Ahn SG Lee HM Cho SH Lee SA Hwang SH Jeong J. Prognostic factors for patients with bone-only metastasis in breast cancer. Yonsei Med J. (2013) 54:1168–77. 10.3349/ymj.2013.54.5.1168 23918566 PMC3743183

[16] Balachandran VP Gonen M Smith JJ DeMatteo RP. Nomograms in oncology: more than meets the eye. Lancet Oncol. (2015) 16:e173–80. 10.1016/S1470-2045(14)71116-7 25846097 PMC4465353

[17] Wang Y Li J Xia Y Gong R Wang K Yan Z. Prognostic nomogram for intrahepatic cholangiocarcinoma after partial hepatectomy. J Clin Oncol. (2013) 31:1188–95. 10.1200/JCO.2012.41.5984 23358969

[18] Fakhry C Zhang Q Nguyen-Tân PF Rosenthal DI Weber RS Lambert L. Development and validation of nomograms predictive of overall and progression-free survival in patients with oropharyngeal cancer. J Clin Oncol. (2017) 35:4057–65. 10.1200/JCO.2016.72.0748 28777690 PMC5736236

[19] Huang YQ Liang CH He L Tian J Liang CS Chen X. Development and validation of a radiomics nomogram for preoperative prediction of lymph node metastasis in colorectal cancer. J Clin Oncol. (2016) 34:2157–64. 10.1200/JCO.2015.65.9128 27138577

[20] Liang W Zhang L Jiang G Wang Q Liu L Liu D. Development and validation of a nomogram for predicting survival in patients with resected non-small-cell lung cancer. J Clin Oncol. (2015) 33:861–9. 10.1200/JCO.2014.56.6661 25624438

[21] Yi M Meric-Bernstam F Kuerer HM Mittendorf EA Bedrosian I Lucci A. Evaluation of a breast cancer nomogram for predicting risk of ipsilateral breast tumor recurrences in patients with ductal carcinoma *in situ* after local excision. J Clin Oncol. (2012) 30:600–7. 10.1200/JCO.2011.36.4976 22253459 PMC3295558

[22] Albert JM Liu DD Shen Y Pan IW Shih YC Hoffman KE. Nomogram to predict the benefit of radiation for older patients with breast cancer treated with conservative surgery. J Clin Oncol. (2012) 30:2837–43. 10.1200/JCO.2011.41.0076 22734034 PMC3410401

[23] Hwang HW Jung H Hyeon J Park YH Ahn JS Im YH. A nomogram to predict pathologic complete response (pCR) and the value of tumor-infiltrating lymphocytes (TILs) for prediction of response to neoadjuvant chemotherapy (NAC) in breast cancer patients. Breast Cancer Res Treat. (2019) 173:255–66. 10.1007/s10549-018-4981-x 30324273

[24] Chang E Mougalian SS Adelson KB Young MR Yu JB. Association between prolonged metastatic free interval and recurrent metastatic breast cancer survival: findings from the SEER database. Breast Cancer Res Treat. (2019) 173:209–16. 10.1007/s10549-018-4968-7 30242577

[25] Ren JX Gong Y Ling H Hu X Shao ZM. Racial/ethnic differences in the outcomes of patients with metastatic breast cancer: contributions of demographic, socioeconomic, tumor and metastatic characteristics. Breast Cancer Res Treat. (2019) 173:225–37. 10.1007/s10549-018-4956-y 30293212 PMC6394580

[26] Soran A Ozmen V Ozbas S Karanlik H Muslumanoglu M Igci A. Randomized trial comparing resection of primary tumor with no surgery in stage IV breast cancer at presentation: protocol MF07-01. Ann Surg Oncol. (2018) 25:3141–9. 10.1245/s10434-018-6494-6 29777404

[27] Pons-Tostivint E Kirova Y Lusque A Campone M Geffrelot J Mazouni C. Survival impact of locoregional treatment of the primary tumor in *de novo* metastatic breast cancers in a large multicentric cohort study: a propensity score-matched analysis. Ann Surg Oncol. (2019) 26:356–65. 10.1245/s10434-018-6831-9 30539492

[28] Xiong Z Deng G Wang J Li X Xie X Shuang Z. Could local surgery improve survival in *de novo* stage IV breast cancer? BMC Cancer. (2018) 18:885. 10.1186/s12885-018-4767-x 30200932 PMC6131766

[29] Fitzal F Bjelic-Radisic V Knauer M Steger G Hubalek M Balic M. Impact of breast surgery in primary etastasized breast cancer: outcomes of the prospective randomized phase III ABCSG-28 POSYTIVE Trial. Ann Surg. (2019) 269:1163–9. 10.1097/SLA.0000000000002771 31082916

[30] Badwe R Hawaldar R Nair N Kaushik R Parmar V Siddique S. Locoregional treatment versus no treatment of the primary tumour in metastatic breast cancer: an open-label randomised controlled trial. Lancet Oncol. (2015) 16:1380–8. 10.1016/S1470-2045(15)00135-7 26363985

[31] Griguolo G Pouderoux S Dieci MV Jacot W Bourgier C Miglietta F. Clinicopathological and treatment-associated prognostic factors in patients with breast cancer leptomeningeal metastases in relation to tumor biology. Oncologist. (2018) 23:1289–99. 10.1634/theoncologist.2018-0200 30120164 PMC6291333

[32] Pece S Disalvatore D Tosoni D Vecchi M Confalonieri S Bertalot G. Identification and clinical validation of a multigene assay that interrogates the biology of cancer stem cells and predicts metastasis in breast cancer: a retrospective consecutive study. Ebiomedicine. (2019) 42:352–62. 10.1016/j.ebiom.2019.02.036 30846393 PMC6491379

[33] Wang J He ZY Dong Y Sun JY Zhang WW Wu SG. The distribution and outcomes of the 21-gene recurrence score in T1-T2N0 estrogen receptor-positive breast cancer with different histologic subtypes. Front Genet. (2018) 9:638. 10.3389/fgene.2018.00638 30619463 PMC6304349

