# Supplementary figures and images for: A Risk Stratification Model for Predicting Overall Survival and Surgical Benefit in Triple-Negative Breast Cancer Patients With de novo Distant Metastasis
Source: Front Oncol. 2020 Jan 24;10:14. doi: 10.3389/fonc.2020.00014 (PMC6992581; doi:10.3389/fonc.2020.00014)

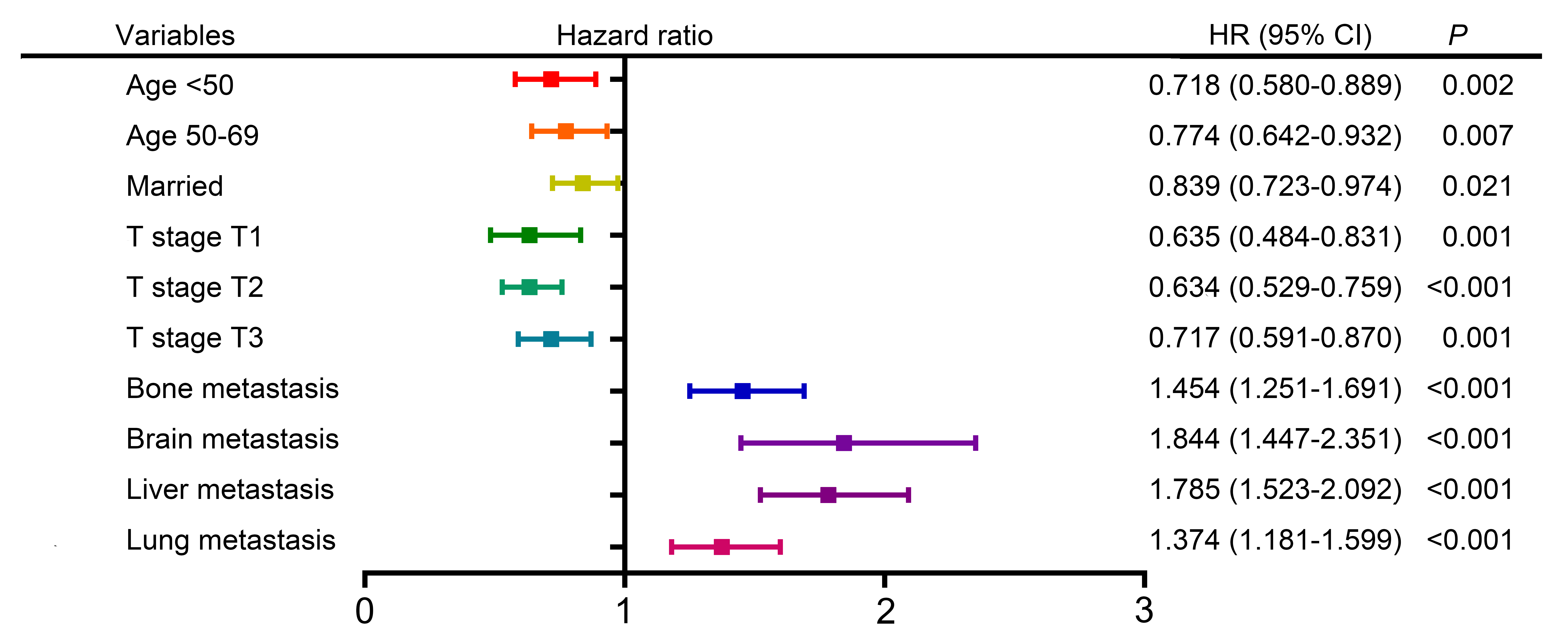

Supplement: Supplementary Figure 1 — Forest plot showing the results of multivariate analysis for breast cancer-specific survival. [file Image_1.TIF]
