# Supplementary material for: A Risk Stratification Model for Predicting Overall Survival and Surgical Benefit in Triple-Negative Breast Cancer Patients With de novo Distant Metastasis
Source: Front Oncol. 2020 Jan 24;10:14. doi: 10.3389/fonc.2020.00014 (PMC6992581; doi:10.3389/fonc.2020.00014)
Supplement: Supplementary Table 1 — Univariate and multivariate analyses for breast cancer-specific survival. [file Table_1.DOCX]

**Supplementary Table 1**. Univariate and multivariate analyses for breast cancer-specific survival.

| Clinicopathological characteristics | Univariable analysis *P* | Multivariable analysis | |
| --- | --- | --- | --- |
|  |  | Hazard ratio (95% CI) | *P* |
| Race | 0.796 |  |  |
| White |  |  |  |
| Black |  |  |  |
| Others |  |  |  |
| Age | 0.015 |  | 0.006 |
| <50 |  | 0.718 (0.580-0.889) | 0.002 |
| 50-69 |  | 0.774 (0.642-0.932) | 0.007 |
| ≥70 |  | Reference |  |
| Marriage | 0.001 |  | 0.021 |
| Married |  | 0.839 (0.723-0.974) | 0.021 |
| Unmarried |  | Reference |  |
| Grade | 0.592 |  |  |
| I |  |  |  |
| II |  |  |  |
| III |  |  |  |
| T stage | <0.001 |  | <0.001 |
| T1 |  | 0.635 (0.484-0.831) | 0.001 |
| T2 |  | 0.634 (0.529-0.759) | <0.001 |
| T3 |  | 0.717 (0.591-0.870) | 0.001 |
| T4 |  | Reference |  |
| N stage | 0.113 |  |  |
| Negative |  |  |  |
| Positive |  |  |  |
| Bone metastasis | <0.001 |  | <0.001 |
| Yes |  | 1.454 (1.251-1.691) | <0.001 |
| No |  | Reference |  |
| Brain metastasis | <0.001 |  | <0.001 |
| Yes |  | 1.844 (1.447-2.351) | <0.001 |
| No |  | Reference |  |
| Liver metastasis | <0.001 |  | <0.001 |
| Yes |  | 1.785 (1.523-2.092) | <0.001 |
| No |  | Reference |  |
| Lung metastasis | <0.001 |  | <0.001 |
| Yes |  | 1.374 (1.181-1.599) | <0.001 |
| No |  | Reference |  |
